# Fabrication and Cell Responsive Behavior of Macroporous PLLA/Gelatin Composite Scaffold with Hierarchical Micro-Nano Pore Structure

**DOI:** 10.3390/nano5020415

**Published:** 2015-03-25

**Authors:** Kedong Song, Lili Ji, Jingying Zhang, Hai Wang, Zeren Jiao, Lim Mayasari, Xiaoyan Fu, Tianqing Liu

**Affiliations:** 1 State Key Laboratory of Fine Chemicals, Dalian R&D Center for Stem Cell and Tissue Engineering, Dalian University of Technology, Dalian 116024, China; E-Mails: liliji@aliyun.com (L.J.); wanghai@dlut.edu.cn (H.W.); jiaozeren@mail.dlut.edu.cn (Z.J.); 2 Medicine Department, Dalian University, Dalian 116622, China; E-Mail: zhangjingying@dlu.edu.cn; 3 Division of Bioengineering, School of Chemical and Biomedical Engineering, Nanyang Technological University, 639798 Singapore, Singapore; E-Mail: mayasari.lim@gmail.com; 4 Department of Obstetrics and Gynecology, First Affiliated Hospital, Dalian Medical University, Dalian 116011, China; E-Mail: fuxiaoyan_111@aliyun.com

**Keywords:** poly-l-lactic acid (PLLA), gelatin, thermally induced phase separation, human adipose derived stem cells, bone tissue engineering

## Abstract

Scaffolds providing a 3D environment which can effectively promote the adhesion, proliferation and differentiation of cells are crucial to tissue regeneration. In this study, the polyllactic acid (PLLA) scaffold with hierarchical pore structural was fabricated via two-step thermally induced phase separation (TIPS). To mimic both physical architecture and chemical composite of natural bone extracellular matrix (ECM), gelatin fibers were introduced into the pores of PLLA scaffolds and formed 3D network structure via TIPS. Human adipose tissue-derived stem cells (ADSCs) were harvested and seeded into PLLA/gel hybrid scaffolds and cultured *in vitro* for biocompatibility assay. The surface morphology, porosity and compressive modulus of scaffolds were characterized by scanning electron microscopy (SEM), density analysis and compression test respectively. The results showed that hybrid scaffolds had high porosity (91.62%), a good compressive modulus (2.79 ± 0.20 MPa), nanometer fibers (diameter around 186.39~354.30 nm) and different grades of pore size from 7.41 ± 2.64 nm to 387.94 ± 102.48 nm. The scaffolds with mild hydrolysis by NaOH were modified by 1-ethyl-3-(3-dimethyl ami-nopropyl) carbodiimide/N-hydroxysuccinimide (EDC/NHS). Gelatin was performed onto PLLA scaffold via TIPS aiming at enhancement cell-material interaction. In comparison with PLLA scaffold, the PLLA/gel scaffold had better biological performance and the mechanical properties because the gelatin fibers homogeneously distributed in each pore of PLLA scaffold and formed 3D network structure.

## 1. Introduction

Bones are rigid and important organs that support and protect the various organs of the body, produce blood cells, store minerals and sever multiple other functions. Bone has the inherent ability to regenerate and spontaneously repair itself; however, the treatment of critical size bones defects caused by trauma, infection, and tumor are still hard to tackle at present [1,2,3]. Autogenous bones, allogenic/allogenous bones, and artificial bone substitutes are now usually used as transplanted materials for bone defects, which have their respective strong and weak points [4,5]. Autogenous bones is considered as the gold standard in the repair of bone defects, but surgeries to get autogenous bones cause a lot of complications, such as pains, blood loss, infection, structure and function destroy of donor sites. In addition, the sources of autogenous bones are relatively less, leading to the limited quantity available. Allogenic/allogenous bones are ready to reject reaction and may lead to infection of pathogens, due to their immunogenicity. Though artificial bone substitutes, such as hydroxylapatite and calcium phosphate, can make up the shortcomings of biological source-derived repairing materials, they are weak in bone induction, their applications are thus limited to bone obturation, bone supporting and bone conduction. Recent development of materials, cytobiology and molecular biology, has made the features and predominaces of tissue or organ reconstruction using tissue engineering methods. The technique of tissue engineering provides a new approach for the reconstruction of bone defects [6].

A typical method of tissue engineering is seeding functional cells into biomimetic scaffolds with special structures and properties to form cell/scaffold constructs *in vitro*, and then transplanted them into the host to repair damaged tissue. Seed cells, scaffolds and growth factors are three key materials of tissue engineering. Adipose-derived stem cells (ADSCs) as kind of seed cells have become the hot point of tissue engineering recently. ADSCs provided advantages such as rich resource, easy collection, mass cells obtained in single harvest, less trauma to organism, *etc.* [7,8]. However, how to obtain enough ADSCs efficiently and induce osteoblast differentiation stably is the principal issue of becoming ideal seed cells for bone tissue engineering.

Fabrication of scaffold materials, as the simulation of extracellular matrix (ECM), is one of the important fields of bone tissue engineering. Two kinds of biomaterials are usually used in tissue engineering, *i.e.*, polyllactic acid (PLLA) and gelatin. PLLA, as thermoplastic aliphatic polyester, is biodegradable and posses biocompatibility [9], which can be derived from renewable resources, such as corn starch. Being able to degrade into innocuous lactic acid, PLLA has been approved as a biomaterial for *in vivo* implantation by American Food and Drug Administration (FDA). However, cell attachment and cell growth on PLLA cannot meet the demand of bone tissue engineering because of its strong hydrophobicity, lack of cell recognition site and insufficient mechanical strength. Gelatin is a partially denatured derivative of collagen [10,11,12,13], which has low immunogenicity, low cost and can be degraded entirely *in vivo*. However, it also has some inherent disadvantages, such as poor mechanical strength and easily hydrolysis. If PLLA and gelatin were combined together to fabricate polymer scaffold, the respective disadvantages of the two materials should be avoided.

In this study, the PLLA scaffold with hierarchical pore structural was fabricated via two-step thermally induced phase separation (TIPS). To mimic both the physical architecture and chemical composite of natural bone ECM, gelatin fibers were introduced into the pores of PLLA scaffolds and formed 3D network structure via TIPS. Finally, hADSCs were harvested and seeded into PLLA/Gel scaffolds and cultured *in vitro*. The biocompatibility of the scaffold and the cell morphology, viability and osteogenic differentiation were all investigated.

## 2. Results and Discussion

### Cellular Morphology

**Figure 1:**
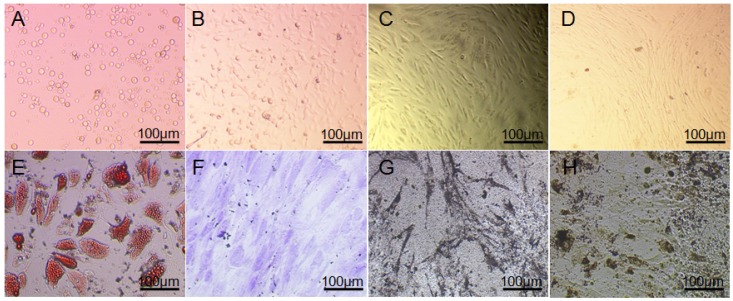
Primary and sub-cultured human adipose tissue-derived stem cells (ADSCs) and their multi-differentiation assays. (**A**) 12 h; (**B**) 3 days; (**C**) 7 days; (**D**) 3 days (P3); (**E**) Stained by oil red O after 2 weeks of differentiating towards adipocytes; (**F**) Stained by toluidine blue after 2 weeks of differentiation towards chondrocytes; (**G**) Alkaline phosphatase (ALP) staining after 2 weeks of differentiation towards osteoblast; (**H**) Von-kossa staining after 4 weeks of differentiation towards osteoblast. Scale bar: **A**–**H**, 100 μm.

As shown in Figure 1A–D, the primary and passaged ADSCs were observed under an inverted microscope in bright field. The cells showed typical morphology of mesenchymal cells, and they spread onto 90%–95% of area of the bottom of T-flasks in every 4–5 days during a passage period, then passage could be carried out. The passaged cells were polygonal, or spindle, dispersing at the bottom of the flasks (Figure 1D), ADSCs appeared to adopt uniform shape with directionality and regularity similar to fibroblast cells with large and oval-shaped nucleus.

A large number of lipid droplets sprang up after two weeks of differentiation, there were a lot of red stained particles in cells after stained by oil red O (Figure 1E), which confirmed the adipogenic differentiation of ADSCs, and secreted matrix could be found around those cells after two weeks of treatment. The differentiated cells were stained positively by toluidine blue which is specific for highly sulfated proteoglycans of cartilage matrices (Figure 1F). ALP assay showed that ADSCs at passage 3 presented a strong alkaline phosphatase activity after 2 weeks of osteogenic induction (Figure 1G). After 4 weeks of incubation, multiple layers of cells emerged. Calcium deposition demonstrated for the nodular deposits was black through von-kossa staining, as shown in Figure 1H.

**Figure 2:**
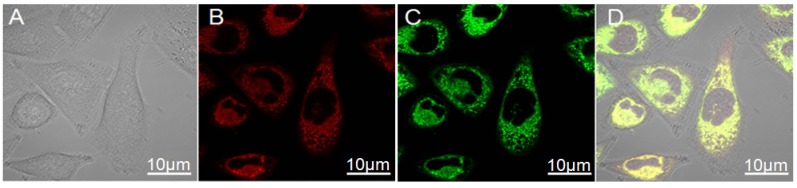
Morphology and cell viability of hADSCs from subcutaneous fat tissue cultured *in vitro*. Single adipose-derived stem cells (ADSCs) at passage 7 (**A**); mitochondria (**B**) of single ADSC stained by MitoTracker^®^ Red CMXRos; cellular nucleus (**C**) of single ADSC stained by SYTO^®^ green fluorescent nucleic acid stains; overlay (**D**) of (**C**) and (**D**). **A**–**D**: ×1000.

After 7 passages *in vitro*, the cell morphology (size and shape) still maintained with only minimal alterations, as shown in Figure 2. Figure 2A–D showed a typical morphology of single ADSC including cellular nucleus and mitochondria stained by SYTO^®^ green fluorescent nucleic acid stains and MitoTracker^®^ Red CMXRos, respectively.

**Figure 3:**
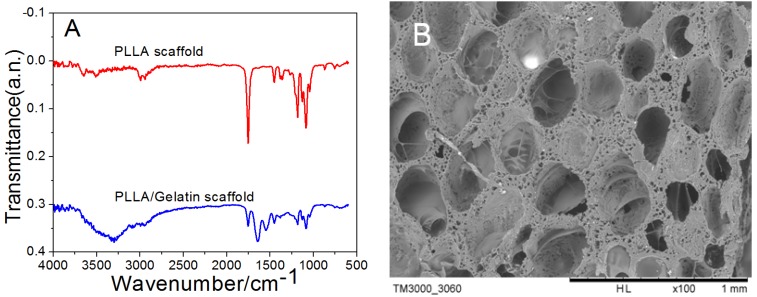
Fourier transform infrared spectroscopy (FTIR) spectra of polyllactic acid (PLLA) and PLLA/gelatin scaffold as well as scanning electron microscopy (SEM) photograph showing pore surface of PLLA scaffold. (**A**) FTIR spectra of PLLA and PLLA/Gelatin scaffolds; (**B**) SEM photograph of PLLA scaffold.

Figure 3A showed fourier transform infrared spectroscopy (FTIR) spectra of polylactic acid scaffold and cross-linked gelatin after polylactic acid scaffold. As shown in IR of polylactic acid, the peak value of 3450 cm^−1^ represented dissociative hydroxyl group due to the presence of water at 1630 cm^−1^, the peak at 2995 cm^−1^, 2944 cm^−1^, 1755 cm^−1^, 1453 cm^−1^ and 1387 cm^−1^ were asymmetric stretching vibration of CH_3_, symmetric stretching vibration of CH_3_, stretching vibration of C=O, asymmetric bending vibration peaks and symmetric bending vibration peaks of CH_3_, respectively. Asymmetric stretching vibration peak of C–O–C and bending vibration peaks in the asymmetric surface of CH_3_ was at 1188 cm^−1^, symmetric stretching vibration peak was at 1090 cm^−1^, bending vibration peaks of C=O was at 766 cm^−1^.

These peaks were consistent with the literature. Gelatin cross-linked poly lactic acid scaffold prepared by phase separation showed many differences. The peak value at 3300–3500 cm^−1^ was higher than that in hydrolysis polylactic acid. The Amino carbonyl stretching vibration absorption peak, N–H bending vibration absorption peak, C–N stretching vibration absorption peak and N–H deformation vibration absorption peak were at 1647 cm^−1^, 1536 cm^−1^, 1447 cm^−1^ and 647 cm^−1^, respectively.

Figure 3B displayed the SEM of PLLA scaffold prepared by secondary phase separation. It was obvious that the pores of PLLA scaffold distributed homogeneously and isotropically, among which the bigger pores were mainly round and elliptical with pore diameter of 191–265 μm. In addition, the pores showed a better connectivity and appear open. The pore wall was rough and showed some clear micro pores with pore diameter of 35.60 ± 14.08 μm, as shown in Figure 3B.

**Figure 4:**
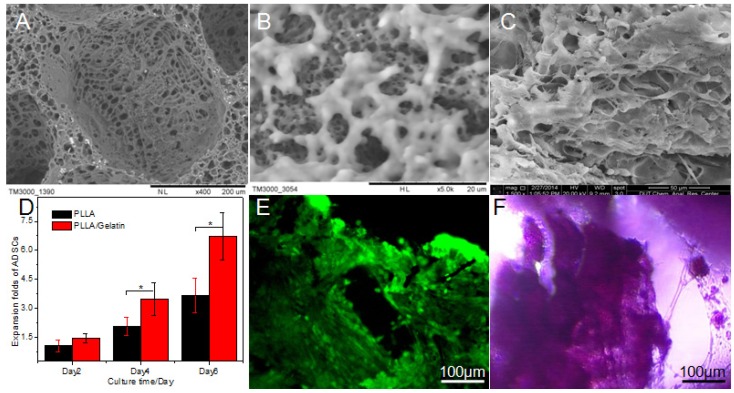
SEM photograph, expansion folds of ADSCs in different scaffolds, representative images for dead/live assay of ADSCs on PLLA/gelatin scaffolds and mineralized tissue formation determined by staining with alizarin red s after 7 days of fabrication. (**A**) Typical morphology of one large pore of PLLA/gelatin scaffold; (**B**) Micro holes of PLLA/gelatin scaffold; (**C**) ADSCs adhered to the surface of PLLA/gelatin scaffold; (**D**) Expansion folds of ADSCs in different scaffolds; (**E**) Fluorescence images for dead/live assay of cell/scaffold constructs; (**F**) Mineralized tissue formation determined by staining with Alizarin Red S. **E**,**F**: ×100.

The scaffolds were put into 4% gelatin solution directly, at vacuum condition, fully infiltrated gelatin into scaffolds, then solvent was extracted at a low temperature, and then the scaffolds were dried for late use. Figure 4A,B showed the SEM photographs showing typical morphology of one large pore and micro holes of PLLA/gelatin scaffold prepared by two-step TIPS and bone. In the modified scaffolds, although the gelatin was evenly spread into the scaffold, the microporous wall of the hole was partly blocked. In addition, gelatin would start to swell after absorbing water, and the swelling of the gelatin would destroy the structure of the scaffolds during their *in vitro* culture with ADSCs. Figure 4A,B were the SEM of PLLA porous scaffolds modified by gelatin in the method of phase separation, gelatin distributed in macropores like membrane sheet or fibrous, and it was uniform distributed on the hole wall, which formed a double scaffold grid structure with the polylactic acid constitutes. The hybrid scaffolds had high porosity (91.62%), a good compressive modulus (2.79 ± 0.20 MPa), nanometer fibers (diameter around 186.39~354.30 nm) and different grades of pore size which large pores with size up to 225.90 ± 34.15 μm and the pore size of microporous from 7.41 ± 2.64 nm to 387.94 ± 102.48 nm.

The ADSCs at passage 7 were innoculated into the sterilized PLLA scaffolds and PLLA/gelatin scaffolds, respectively. With increasing time of culture, ADSCs showed good growth status in different kinds of scaffolds (Figure 4C). Cell proliferation rate from different scaffolds were all detected at the first 2 days, 4 days and 6 days, respectively (Figure 4D). Cellular growth of ADSCs on PLLA/gelatin scaffolds was better than that in PLLA scaffolds and hydrolyzed PLLA scaffolds. For one thing, the addition of gelatin could improve the biocompatibility of pure PLLA scaffolds. For another, gelatin inside the scaffold absorbed water so that nutrients could infiltrate into internal scaffolds. The PLLA/gelatin scaffolds prepared by phase separation were superior to other prepared scaffolds, but they were not statistically significant.

Figure 4E showed live/dead staining of different culture days with ADSCs in PLLA/gelatin hybrid scaffolds. Judging from the results of staining, after inoculated one day, the cells on the scaffold were more uniform distributed, and began to attach to the walls and holes of hybrid scaffolds. On the third day, the proliferated cells could be clearly seen. On the seventh day, as shown in Figure 4C, cells grew and connected with each other to form lamellar structure, and only few cells were dead (Figure 4E). Moreover, the cells fully extended into the scaffolds. Mineralized tissue formation was determined by staining with alizarin red s that gives red staining with calcium salt crystallization (Figure 4F). It confirmed that the hybrid scaffolds had a very good biocompatibility and were conducive to cell growth and adhesion.

## 3. Experimental Section

### 3.1. Isolation and Culture of hADSCs

Adipose tissue was obtained from abdominal adipose of an adult female with informed consent after abdominal liposuction by our improved method [11]. Adipose tissue was digested by mixture of 0.25% trypsin and 0.1% of type I collagenase (Sigma, St. Louis, MO, USA), the bottom layer including mononuclear cells was extracted, and digestion was terminated with h-glucose DMEM (Sigma) containing 15% fetal bovine serum (FBS, Gibco, Waltham, MA, USA). The remnant adipose tissue was digested repeatedly for three times. Cell pellets were afterward suspended in medium (DMEM + 10% FBS). Cells were cultured at 37 °C and 5.0% CO_2_ in a humidified incubator, with full media replacement every 3 days. When the cells reached 85% confluence, they were digested by the mixture of 0.25% trypsin plus 0.04% EDTA (Shanghai Reagent, Shanghai, China) and passaged for late use.

### 3.2. Cell Morphology and Multiple Differentiation Potential of ADSCs

Cellular growth and morphology of ADSCs were observed under an inverted microscope (Olympus, Tokyo, Japan) in a bright field mode and a confocal laser scanning microscopy (CLSM, Olympus). Hematoxylin-eosin (HE) staining described by Song *et al.* [12] was also applied in this study for assay of cell morphology.

**Osteogenic Differentiation:** ADSCs collected at passage 7 were seeded into 24-well plates at 5 × 10^4^ cells/mL. Six holes were seeded for alkaline phosphatase (ALP) staining, another six holes for von Kossa staining, and the remaining holes were served as negative control. Culture media were fully replaced every 3 days with osteogenic induced media while the cells began to adhere to the subface. After 2 and 4 weeks of culture with induced media, the cells were stained with ALP and von Kossa, respectively.

**Chondrogenic and Adipogenic Differentiations:** The samples were prepared as above method and cultured in an incubator at 37 °C with a 100% humidified chamber and 5% CO_2_. Culture media were fully replaced every 3 days by chondrogenic and adipogenic differentiation media after the cells began to adhere to the holes, respectively. The morphological changes of cells were observed under a microscope. The samples were stained by oil red and toluidine blue [13] after 2 weeks of inductions.

### 3.3. Laser Confocal Microscopy Examination

The mitochondria and cellular skeletons of ADSCs were marked with MitoTracker^®^ Red CMXRos; cellular nucleus and SYTO^®^ green fluorescent nucleic acid stains respectively, and then were observed after being incubated at 37 °C for 45 min [14,15].

### 3.4. Preparation of PLLA Scaffold

PLLA solution was prepared with mass fraction of 6% by dissolving PLLA into 1, 4-dioxane solvent/water system (87/13, wt/wt). The prepared solution was then dissolved into homogenous solution by magnetic stirring in a water bath at 60 °C. After that, 5 mL homogenous solution was put into the precooled mould with initial gelling point at 4 °C and gelling time of 5 min. The resulting gel was then kept at −20 °C for 4 h, the generating sample was put into liquid nitrogen for final quenching. After that, the solvent would be substituted by the method of frozen extraction using the 80% ethanol water at −20 °C for 1 day and brine at 0 °C for 2 day. The sample was then dried at room temperature for 1 day. The prepared scaffold is white with rough surface and spongy porous structure.

### 3.5. Modification of PLLA Scaffold

PLLA scaffold was cut into size of 0.5 cm × 0.5 cm × 0.5 cm with surgical knife blade, and then they were put into 0.2% NaOH solution. They were absorbed under the vacuum and held at room temperature for 15 min after restoring normal pressure. Then, the removed scaffolds were centrifuged under 1000 rpm for 5 min and then embathed in PBS for 10 min, rinsed for twice using distilled water. Afterwards, the treated scaffold were immersed into 1% EDC and then were moved into 4% gelatin alcohol/water (*v*:*v* = 1:1) after rinsing twice with distilled water. The solution within the scaffold was absorbed under the vacuum and the gelatin will be fully infiltrated into the scaffold after recovering normal pressure. Finally, they were prepared by induced phase separation at −70 °C for at least 4 h and then quenched using liquid nitrogen, and then were dried under vacuum after extracted with alcohol at low temperature for 2 days.

### 3.6. IR Spectra Analysis

Different scaffolds were tested using a TENSOR27 FTIR spectrometer (Bruker, Erkrath, Germany). The samples were dispersed uniformly in the KBr slice using KBr tabletting, after which whether the PLLA and gelatin were interacted or not would be analyzed according to the variation of the characteristic absorption peak in the spectrogram.

### 3.7. Morphology Observation and Detection by SEM

In order to dry the prepared PLLA scaffolds and hybrid scaffold for SEM analysis, these were fixed and dehydrated for 15 min each time in increasing concentrations of ethanol in water (50%, 70%, 80%, 90% and 100%); afterwards, they were immediately dried with an HMDS solution for 5 min. Following this, the samples were coated with platinum, palladium and carbon and observed under SEM at 40 kV [2,3].

Ten pores on the surface of PLLA/gelatin scaffolds on SEM were randomly selected for detecting the diameters; and the pore size distributions were then calculated and expressed as mean ± SD.

### 3.8. Statistical Analysis

All experiments were performed in triplicate. Results are expressed as the mean ± SD. The statistical significance of differences within each group was evaluated by one-way analysis of variance (1-way ANOVA) using the software Origin7.0 (OriginLab Corporation, Northampton, MA, USA).

## 4. Conclusions

PLLA scaffolds which were prepared by way of two-step phase separation have a good porosity and mechanical properties with open pore structures and pore diameters in different levels. The scaffolds were hydrolyzed mildly and cross-linked with EDC. Gelatin could be integrated into PLLA scaffolds through TIPS. The grid-like distribution of gelatin on the pore walls of scaffolds not only increased the specific surface area and hydrophilic properties of scaffolds, but also notably improved their mechanical properties and protein adsorption capacity. After inoculated to the composite scaffolds for culture, the ADSCs can proliferate numerously with a lamellar growth, which indicates that the scaffolds have good cell compatibility, and provides a good prospect for the further application of the scaffolds.
